# Association between preoperative serum Cystatin‐C levels and postsurgical oncological prognosis in patients with PRCC: A retrospective cohort study

**DOI:** 10.1002/cam4.4731

**Published:** 2022-04-05

**Authors:** Shiyang Lu, Shijie Li

**Affiliations:** ^1^ Department of Urology Shengjing Hospital of China Medical University Shenyang PR China

**Keywords:** biomarker, cystatin‐C, papillary renal cell carcinoma, prognostic factors, retrospective study

## Abstract

**Objective:**

Cystatin‐C (Cys‐C) is a predictor of several malignancies. However, whether Cys‐C levels predict prognosis in patients with papillary renal cell carcinoma (PRCC) remains uncertain. The aim of this study was to assess the correlation between Cys‐C and clinical outcomes in patients with PRCC.

**Methods:**

The medical records of 137 patients with PRCC who underwent surgery at our institution from January 2008 to December 2020 were retrospectively analyzed. Data were divided into two subgroups based on cutoff values and the relationship between the Cys‐C group and their clinical outcomes was assessed.

**Results:**

By the last follow‐up, 62 patients had died of various causes, 53 of whom died from PRCC. Sixty patients suffered recurrence or metastasis during follow‐up. Based on the cutoff value, the patients were divided into two groups: low Cys‐C group (Cys‐C < 1.25 mg/L, *n* = 92) and high Cys‐C group (Cys‐C ≥ 1.25 mg/L, *n* = 45). Pathological classification and serum Cys‐C levels were shown to be independent prognostic factors affecting clinical outcomes, according to multivariate Cox regression analysis (*p* < 0.05). After adjusting the Cox proportional hazards model, the risk of death was elevated in the high Cys‐C group. The results of the area under the curve for time‐dependent receiver operating characteristics analysis indicated that Cys‐C is a stable and reliable prognostic biomarker for predicting survival in patients with PRCC. Forest plots, constructed to better reflect the comparison of hazard ratios between the two groups, confirmed that Cys‐C levels were significantly associated with worsening overall survival.

**Conclusion:**

This study is the first to examine the relationship between preoperative serum Cys‐C levels and prognostic overall survival in patients with PRCC. Cys‐C may be a useful biomarker for preoperative screening of high‐risk patients who may require adjuvant therapy.

## Graphical abstract

1. This study is the first to examine the relationship between Cys‐C levels and OS in patients with PRCC. 2. Cys‐C may be a useful preoperative screening biomarker 3. Elevated Cys‐C levels were significantly associated with worsening OS.

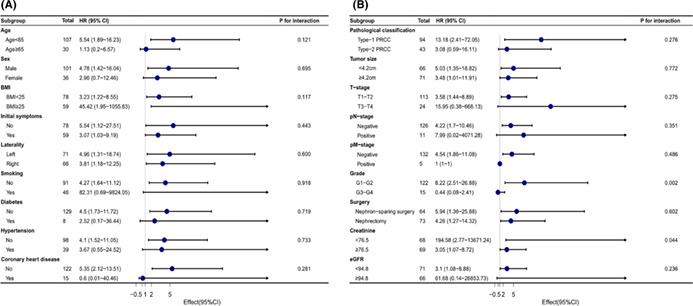

## 1 INTRODUCTION

Papillary renal cell carcinoma (PRCC) is the second most common type of renal cell carcinoma (RCC).^1^ Histologically, PRCC is characterized by the presence of a vascular core and a papillary arrangement of tumor cells; it is divided into type 1 and type 2 subtypes depending on histological and genetic characteristics.^2^, ^3^ Although surgery remains the primary treatment for localized disease, approximately 40% of surgically resected patients have experienced tumor recurrence. Cytotoxic chemotherapy and immunotherapy have been widely used in the past for treating patients diagnosed with locally advanced or metastatic PRCC, but with limited efficacy.^4^ To design an optimum treatment strategy, it is important to determine the potential outcome of patients with PRCC at an early stage.

Although the TNM staging system is widely used for cancer treatment evaluation, it does not effectively predict survival outcomes.^5^, ^6^, ^7^ As these prognostic factors are not well established, clinicians have now been focusing on novel markers, including laboratory and clinical indicators. Studies have shown that laboratory indicators, such as platelets, neutrophil lymphocyte ratio, plasma fibrinogen, D‐dimer, and serum albumin/globulin ratio are effective in assessing the prognosis of RCC.^8^, ^9^, ^10^, ^11^, ^12^ However, most of the studied biomarkers cannot be widely used in the clinic because of their taught difficult measurements and expensive price, so we need to focus on “new” methods such as preoperative serum cystatin‐c levels.

Serum cystatin‐C (Cys‐C) is an endogenous marker of glomerular filtration rate (GFR), which is more sensitive than serum creatinine. Previous studies have shown that abnormal serum Cys‐C levels can be used as a prognostic and diagnostic indicator for breast cancer, myeloma, colon cancer, renal cell carcinoma, and non‐Hodgkin's B‐cell lymphoma.^13^, ^14^, ^15^, ^16^, ^17^ Notably, high preoperative Cys‐C levels are associated with poor survival in patients with RCC.^17^, ^18^ However, the majority of the cases in this study were clear cell RCC subtypes, with a small proportion of PRCC (6.2%). To the best of our knowledge, no relevant studies, to date, have confirmed that the prognosis of PRCC is associated with Cys‐C levels. Therefore, the purpose of this study was to retrospectively evaluate the implications of using serum Cys‐C levels for the prognosis of patients with PRCC undergoing surgical treatment.

## 2 MATERIALS AND METHODS

### 2.1 Patient selection

**FIGURE 1:**
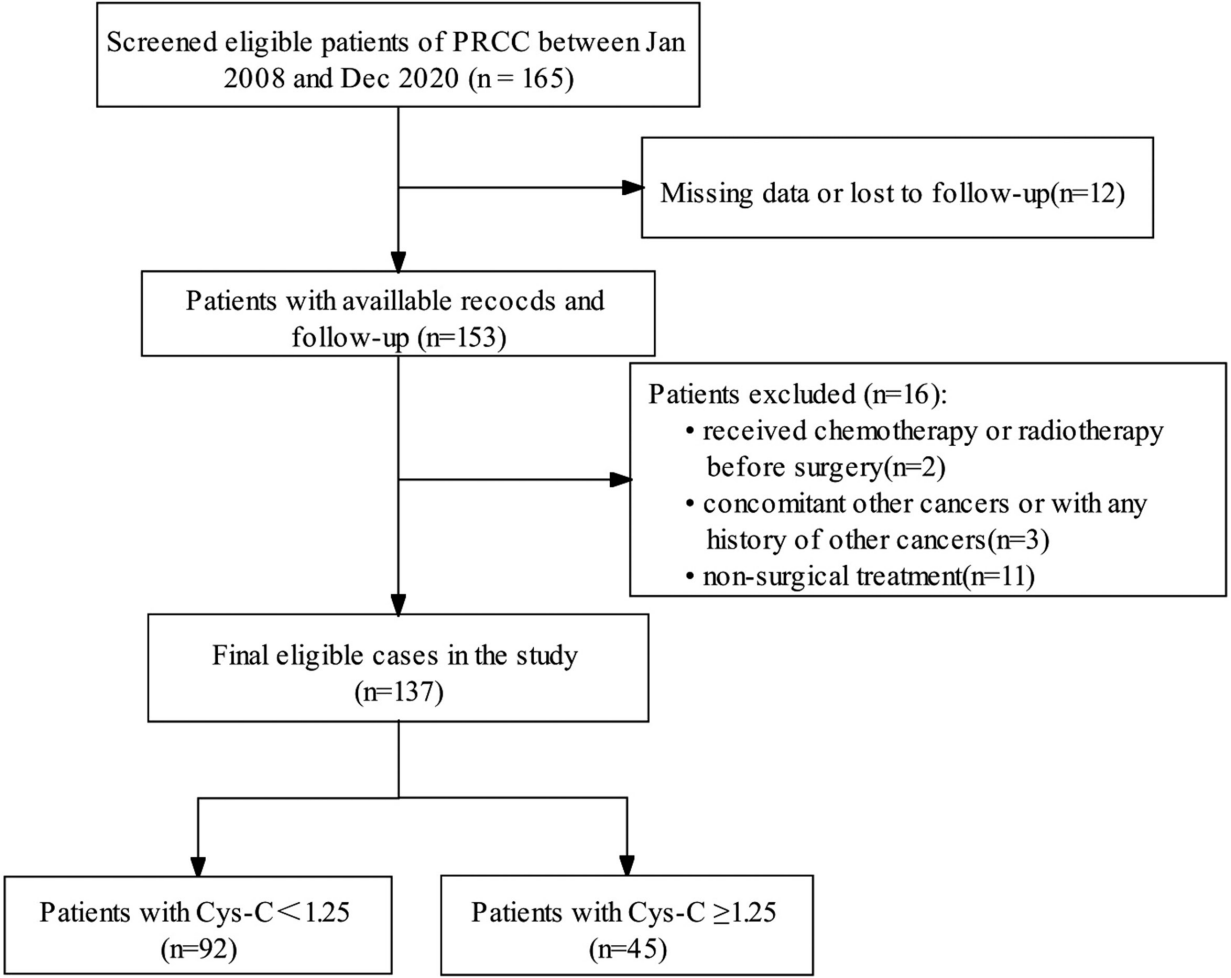
Patient selection flowchart

The medical records of 165 patients with PRCC, who underwent surgical treatment at our hospital from January 2008 to December 2020, had been retrospectively analyzed. The inclusion criteria were as follows: (1) pathologically confirmed PRCC, (2) the surgical approach was radical or partial nephrectomy, (3) no preoperative adjuvant therapy was administered, and (4) the medical records were complete. The exclusion criteria for data collection were as follows: (1) concomitant other cancer or a history of other cancers, (2) no treatment with partial or total nephrectomy, (3) patients received radiotherapy or chemotherapy before surgery, and (4) incomplete clinicopathological features and follow‐up data. After screening, the final cohort for further analysis included 137 individuals (Figure 1). This study was approved by the Ethics Committee of Shengjing Hospital of China Medical University. Because it was a retrospective cohort study, the requirement for obtaining informed consent from the patients was waived by the Ethics Committee. The clinical research registry number is 2021PS692K.

### 2.2 Data collection and variables

The laboratory and clinicopathological data were collected in a nonselective and continuous manner from a electronic medical record system of our hospital, which is the single database center. Covariables contained demographic and clinicopathological data, as well as variables known to affect either Cys‐C or clinical outcomes. The 2016 World Health Organization Classification of Tumors of the Urinary System and the American Joint Committee of Cancer TNM staging classification (8th edition) were used to evaluate the grade and tumor stage, respectively.^19^, ^20^ Other tests included chest x‐rays/computed tomography (CT), routine laboratory tests, bone scans, and ultrasound to initially screen for distant metastases and, if necessary, positron emission tomography (PET)‐CT. The Hospital Information System database was used to retrieve the preoperative serum Cys‐C values of the patients from the results of routine blood tests performed within a period of 30 days prior to surgery. The variables used to establish the fully adjusted model were sex, body mass index (BMI), age, initial symptoms, laterality, smoking history, diabetes, hypertension, coronary heart disease, pathological classification, T‐stage, tumor size, distant metastasis, lymph node status, tumor grade, surgery, serum Cys‐C levels, estimated glomerular filtration rate (eGFR), and serum creatinine levels.

### 2.3 Patient follow‐up

Follow‐up assessments included laboratory tests, physical examinations, and radiological examinations, in accordance with the clinical practice guidelines of the National Comprehensive Cancer Network (NCCN). Physical and laboratory examinations were performed every 3 months, and magnetic resonance imaging (MRI) or enhanced CT was examined every 3–6 months. Local recurrence or distant metastasis was diagnosed by CT, bone scan, MRI, PET‐CT, and other imaging techniques. The primary outcome was overall survival (OS); secondary outcomes included progression‐free survival and cancer‐specific survival.

### 2.4 Statistical analysis

Continuous variables and categorical variables were expressed as median and interquartile range (IQR), and frequencies (percentage), respectively. The X‐tile software (version 3.6.1) was used to calculate the optimal cutoff point of preoperative Cys‐C levels, based on which we could treat continuous Cys‐c as a categorical variable and classify the entire cohort into two groups. The Mann–Whitney *U* test and chi‐square test were used to test the correlation between the groups of variables. Initially, we developed Cox proportional risk models of univariate and multifactor analysis. In this study, covariates were included in a Cox regression model; by comparing regression coefficients, we assessed the impact of confounding factors. The models we set up were as follows: covariates unadjusted model; model 1–adjusted for age, BMI, sex only; model 2–adjusted for age, BMI, sex, initial symptoms, laterality, smoking history, diabetes, hypertension, and coronary heart disease; and model 3–adjusted for all covariates. The area under the curve (AUC) and time‐dependent relative operating characteristic (ROC) curve analysis were also performed to assess the predictive accuracy of Cys‐C for survival prediction. The Kaplan–Meier curve (log rank test) was adopted to analyze the impact of Cys‐C on survival outcomes. A subgroup analysis using the Cox proportional risk model assessed the survival differences between the two Cys‐C groups. Further, a forest plot was constructed to better reflect the comparison of hazard ratios (HRs) between the two groups, in each subgroup.

The statistical software package R v.4.0.2 (http://www.R‐project.org, The R Foundation) and Free Statistics software versions 1.3, were used for statistical analyses. All tests were two‐tailed. Statistical significance was set at *p* < 0.05.

## 3 RESULTS

### 3.1 Baseline Clinicopathologic Characteristics

**FIGURE 2:**
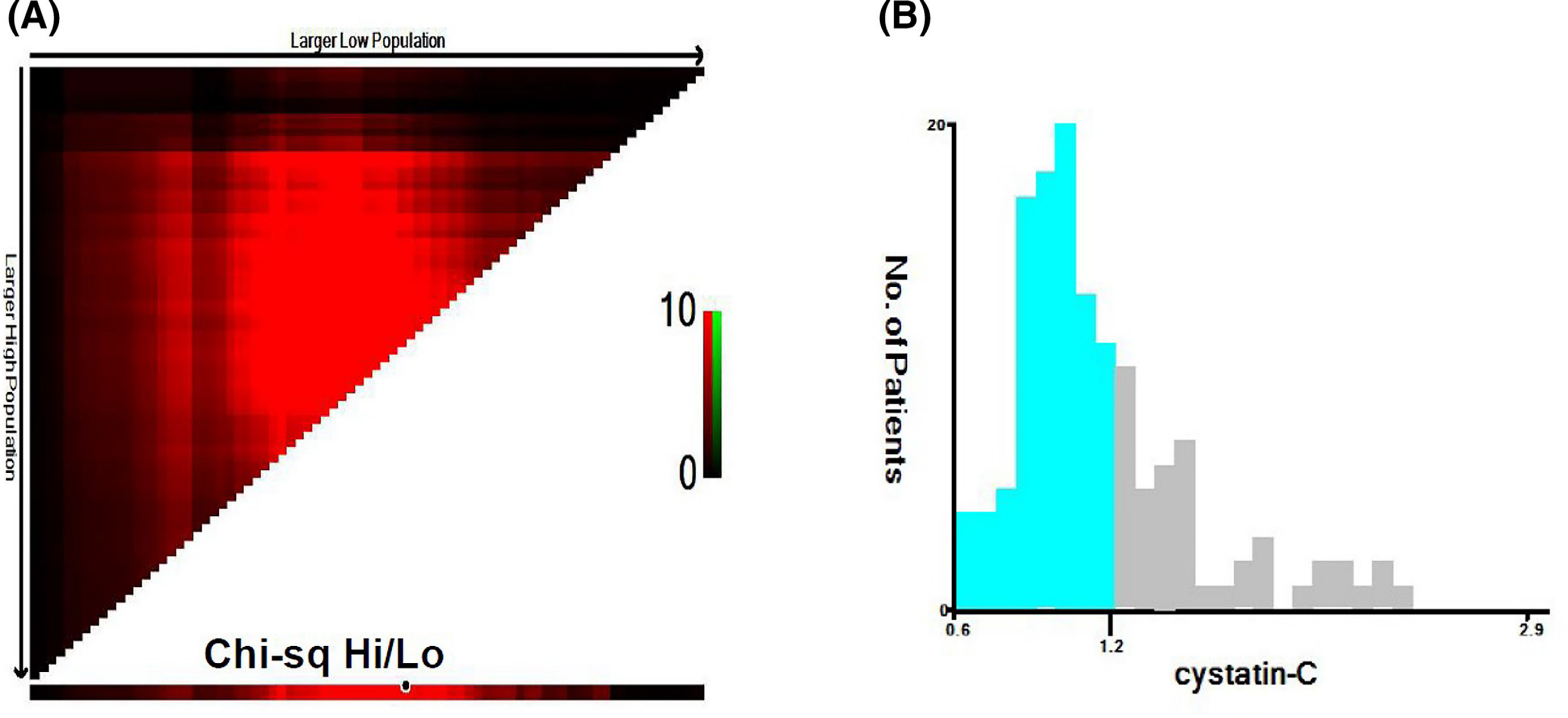
X‐tile analyses determined the optimal cutoff value of Cystatin‐C for overall survival based on patient data. The optimal cutoff values are displayed in histograms of the entire cohort. The optimal cutoff value was 1.25 mg/L

A retrospective analysis was performed using the data from 137 patients with PRCC, who had received surgical treatment at Shengjing Hospital of China Medical University from 2008 to 2020. The mean follow‐up time was 24 months. At the final follow‐up, 62 patients died due to various causes, 53 of whom died of RCC. Sixty patients experienced recurrence or metastasis at follow‐up. This study's optimal cutoff value for Cys‐C, obtained by X‐tile software, was 1.25 mg/L (Figure 2). Patients were then classified into the following two groups: low Cys‐C group (Cys‐C < 1.25 mg/L, *n* = 92) and high Cys‐C group (Cys‐C ≥ 1.25 mg/L, *n* = 45). The correlation between various levels of Cys‐C and clinicopathological features is shown in Table 1. There were no significant differences between the two groups in terms of smoking, BMI, laterality, sex, diabetes, hypertension, coronary artery disease, tumor size, T‐stage, surgery approach, and lymph node status (*p* > 0.05). In the high Cys‐C group, patients tended to be older and with a higher pathological grade (*p* = 0.037) than those in the low Cys‐C group. In addition, patients with high levels of Cys‐C developed distant metastases (*p* = 0.040), most of which were pathological type‐2 PRCC (*p* = 0.035).

**TABLE 1:** The relationship between cystatin‐C groups and clinicopathological parameters in the present cohort (*n* = 137)

Characteristics	Total (*n* = 137)	Cys‐C <1.25 (*n* = 92)	Cys‐C ≥1.25 (*n* = 45)	*p*‐value
Age, year, Median (IQR)	58.0 (49.0, 64.0)	53.0 (47.0, 63.0)	63.0 (57.0, 69.0)	**<0.001**
Sex, *n* (%)				
Male	101 (73.7)	65 (70.7)	36 (80)	0.337
Female	36 (26.3)	27 (29.3)	9 (20)	
BMI, kg/m^2^, Median (IQR)	24.8 (22.1, 27.7)	24.7 (22.5, 27.7)	24.8 (21.4, 27.0)	0.371
Initial symptoms, *n* (%)				
No	78 (56.9)	60 (65.2)	18 (40)	**0.009**
Yes	59 (43.1)	32 (34.8)	27 (60)	
Laterality, *n* (%)				
Left	71 (51.8)	47 (51.1)	24 (53.3)	0.948
Right	66 (48.2)	45 (48.9)	21 (46.7)	
Smoking, *n* (%)				
No	91 (66.4)	62 (67.4)	29 (64.4)	0.880
Yes	46 (33.6)	30 (32.6)	16 (35.6)	
Diabetes, *n* (%)				
No	129 (94.2)	86 (93.5)	43 (95.6)	1.000
Yes	8 (5.8)	6 (6.5)	2 (4.4)	
Hypertension, *n* (%)				
No	98 (71.5)	69 (75)	29 (64.4)	0.278
Yes	39 (28.5)	23 (25)	16 (35.6)	
Coronary heart disease, *n* (%)				
No	122 (89.1)	84 (91.3)	38 (84.4)	0.252
Yes	15 (10.9)	8 (8.7)	7 (15.6)	
Pathological classification, *n* (%)				
Type 1	94 (68.6)	69 (75)	25 (55.6)	**0.035**
Type 2	43 (31.4)	23 (25)	20 (44.4)	
Tumor size, cm, Median (IQR)	4.2 (2.7, 6.0)	4.0 (2.7, 6.0)	4.7 (3.0, 6.0)	0.260
T‐stage, *n* (%)				
T1‐T2	113 (82.5)	78 (84.8)	35 (77.8)	0.439
T3‐T4	24 (17.5)	14 (15.2)	10 (22.2)	
Lymph node status, *n* (%)				
Negative	126 (92.0)	87 (94.6)	39 (86.7)	0.177
Positive	11 (8.0)	5 (5.4)	6 (13.3)	
Distant metastasis, *n* (%)				
No	132 (96.4)	91 (98.9)	41 (91.1)	**0.040**
Yes	5 (3.6)	1 (1.1)	4 (8.9)	
Tumor grade, *n* (%)				
G1‐G2	122 (89.1)	86 (93.5)	36 (80)	**0.037**
G3‐G4	15 (10.9)	6 (6.5)	9 (20)	
Surgery, *n* (%)				
Nephron‐sparing surgery	64 (46.7)	40 (43.5)	24 (53.3)	0.366
Nephrectomy	73 (53.3)	52 (56.5)	21 (46.7)	
Serum creatinine, μmol L^−1^, Median (IQR)	76.5 (64.1, 87.5)	69.5 (59.2, 77.2)	91.6 (80.9, 121.5)	**<0.001**
eGFR (ml min^−1^ per 1.73 m^2^), Median (IQR)	94.8 (79.3, 109.9)	103.0 (91.9, 114.0)	68.5 (53.5, 83.8)	**<0.001**
Cys‐C, Median (IQR)	1.1 (1.0, 1.3)	1.0 (0.9, 1.1)	1.5 (1.3, 1.8)	**<0.001**

Abbreviations: BMI, body mass index; Cys‐C, cystatin‐C; eGFR, estimated glomerular filtration rate; IQR, interquartile range.

Statistically significant values at *p* < 0.05 are shown in bold.

### 3.2 Univariate and multivariate Cox regression analysis

Univariate and multivariate Cox regression analyses were used to explore independent risk factors for OS (Table 2). Univariate cox proportional hazards model confirmed that BMI, initial symptoms, diabetes, pathological staging, tumor grade, serum creatinine, eGFR, and Cys‐C levels were associated with poor OS (*p* < 0.1). Using the significant prognostic factors identified in the univariate analysis to determine a multivariate Cox regression analysis, it was determined that pathological classification and Cys‐C levels were independent prognostic factors affecting clinical outcome (*p* < 0.05).

**TABLE 2:** Prognostic factors for patients' survival in univariate and multivariate Cox regression analyses

Covariates	Univariate	Multivariate		
	HR (95% CI)	*p*‐value	HR (95% CI)	*p*‐value
Age (year)	1.02 (0.99, 1.05)	0.203		
Sex (female vs. male)	1.89 (0.73, 4.91)	0.191		
BMI (kg/m^2^)	0.88 (0.78, 0.99)	**0.040**	0.89 (0.78–1.02)	0.095
Initial symptoms (yes vs. no)	3.08 (1.11, 8.51)	**0.030**	0.78 (0.23–2.64)	0.694
Laterality (right vs. left)	1.25 (0.52, 3.05)	0.616		
Smoking: (yes vs. no)	0.76 (0.28, 2.11)	0.603		
Diabetes: (yes vs. no)	3.89 (1.09, 13.86)	**0.036**	2.85 (0.46–17.56)	0.260
hypertension: (yes vs. no)	1.14 (0.41, 3.17)	0.800		
Coronary heart disease (yes vs. no)	1.05 (0.31, 3.6)	0.939		
Pathological classification:(type 2 vs. 1)	3.57 (1.4, 9.08)	**0.008**	9.63 (2.51–36.95)	**0.001**
Tumor size (cm)	0.98 (0.87, 1.11)	0.789		
T‐stage (T3‐T4 vs. T1‐T2)	0.36 (0.08, 1.58)	0.178		
Lymph node status (positive vs. negative)	1.38 (0.31, 6.06)	0.673		
Distant metastasis (yes vs. no)	0.66 (0.09, 5.09)	0.693		
Tumor grade (G3‐G4 vs. G1‐G2)	5.32 (2.13, 13.33)	**< 0.001**	2.23 (0.79–6.23)	0.128
Surgery (nephrectomy vs. nephron‐sparing surgery)	1.09 (0.45, 2.65)	0.841		
Serum creatinine (μmol L^−1^)	1.01 (1.00, 1.01)	**0.070**	0.99 (0.97–1.02)	0.563
eGFR (ml min/L per 1.73 m^2^)	0.98 (0.97, 1)	**0.027**	0.99 (0.96–1.02)	0.561
Cys‐C (continuous)	4.28 (1.8, 10.2)	**0.001**	5.59 (1.03–30.31)	**0.046**

Abbreviations: BMI, body mass index; Cys‐C, cystatin‐C; eGFR, estimated glomerular filtration rate; IQR, interquartile range.

Statistically significant values at *p* < 0.05 are shown in bold.

### 3.3 Association of serum Cys‐C levels with OS

We investigated the association between serum Cys‐C levels and OS by constructing unadjusted and adjusted Cox proportional hazards models (Table 3). In the unadjusted model, the HR for the effect of high serum Cys‐C levels on OS was 7.0, with a 95% confidence interval (95% CI) of 2.3–21.2 (*p* < 0.001). In adjusted model 1, after adjustment for age, sex, and BMI, high serum Cys‐C levels remained an independent risk factor for OS (HR = 7.5, 95% CI: 2.2–25.2, *p* = 0.001). In adjusted model 2, after adjusting for age, sex, BMI, initial symptoms, laterality, smoking history, diabetes, hypertension, and coronary heart disease, the HR for the effect of high serum Cys‐C levels on OS was 9.5 (95% CI: 2.6–34.9, *p* < 0.001). In fully adjusted model 2, after adjusting for age, sex, BMI, initial symptoms, laterality, smoking history, diabetes, hypertension, coronary heart disease, pathological classification, tumor size, tumor grade, lymph node status, T‐stage, distant metastasis, surgery, serum creatinine levels, and eGFR, high serum Cys‐C levels remained independently associated with OS (HR = 17.2, 95% CI: 2.9–102.4, *p* = 0.002).

**TABLE 3:** Multiple Cox regression analysis of Cys‐C in patients with PRCC

Cys‐C	Non‐adjusted	*p*‐value	Adjust I	*p*‐value	Adjust II	*p*‐value	Adjust III	*p*‐value
Continuous	4.3 (1.8, 10.2)	**0.001**	4.2 (1.4, 12.7)	**0.011**	5.1 (1.5, 17.7)	**0.011**	1.2 (0.2, 7.1)	0.854
Group								
Cys‐C<1.25	1 (reference)		1 (reference)		1 (reference)		1 (reference)	
Cys‐C≥1.25	7.0 (2.3, 21.2)	**<0.001**	7.5 (2.2, 25.2)	**0.001**	9.5 (2.6, 34.9)	**<0.001**	17.2 (2.9, 102.4)	**0.002**

*Note*: Non‐adjusted model adjusted for: None.

Adjust I model adjusted for: age, sex, BMI.

Adjust II model adjusted for: age, sex, BMI, initial symptoms, laterality, smoking history, diabetes, hypertension, coronary heart disease.

Adjust III model adjusted for: age, sex, BMI, initial symptoms, laterality, smoking history, diabetes, hypertension, coronary heart disease, pathological classification, tumor size, T‐stage, lymph node status, distant metastasis, tumor grade, surgery, serum creatinine, eGFR.

Abbreviations: BMI, body mass index; Cys‐C, cystatin‐C; eGFR, estimated glomerular filtration rate.

Statistically significant values at *p* < 0.05 are shown in bold.

**FIGURE 3:**
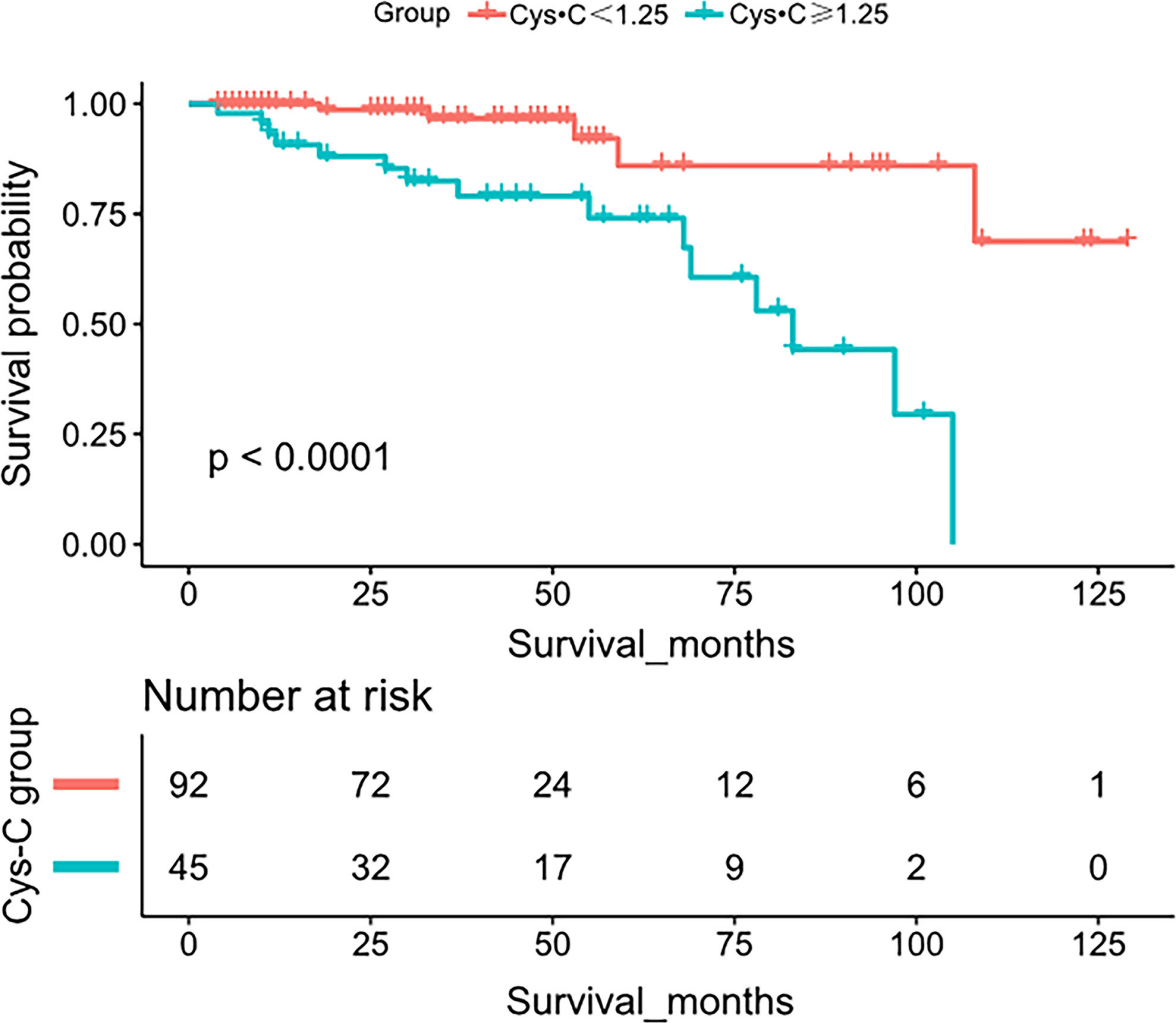
Kaplan–Meier curves for OS of patients with PRCC based on Cys‐C stratification results. Kaplan–Meier analyses showed that OS times were shorter in patients with high levels of serum Cys‐C than in those with low levels of serum Cys‐C. PRCC, Papillary renal cell carcinoma; OS, overall survival; Cys‐C, Cystatin‐C

**FIGURE 4:**
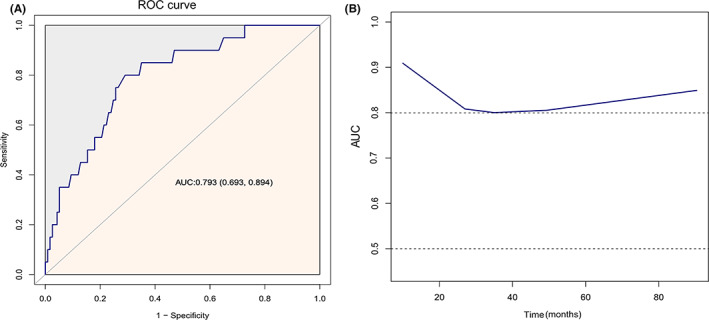
(A) Receiver operating characteristic (ROC)curves of Cys‐C concerning OS. (B) Time‐dependent area of Cys‐C under the characteristic curve (AUROC) for overall survival after the start of follow‐up. At each time point, the (AUROC) was more than 0.8

In addition, Kaplan–Meier analyses showed that OS times were shorter in patients with high levels of serum Cys‐C than in those with low levels of serum Cys‐C (Figure 3). As shown in Figure 4A, ROC analysis showed that AUC was 0.793 (95% CI: 0.693–0.894), suggesting the prediction ability of Cys‐C for predicting survival. The AUC for time‐dependent ROC performed well, indicating that Cys‐C levels were a promising prognostic biomarker and offered superior reliability and stability to predict survival in patients with PRCC (Figure 4B).

### 3.4 Subgroup analyses

**FIGURE 5:**
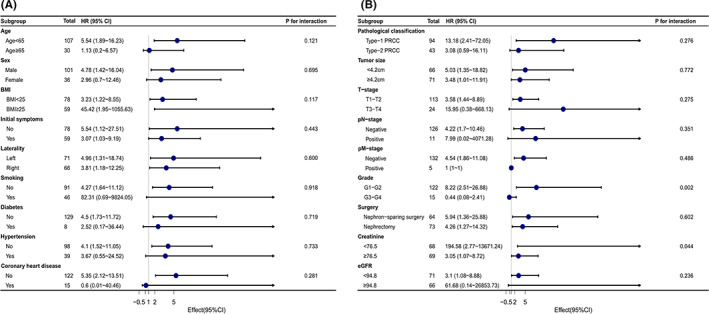
Forest plot for presenting the relationship of the hazard ratio of overall survival and Cys‐C in patients with PRCC. Elevated Cys‐C level was found to be significantly associated with poor OS in almost all subgroups. PRCC, Papillary renal cell carcinoma; Cys‐C, Cystatin‐C

To investigate the effect of serum Cys‐C levels on prognosis in different subgroups with PRCC, we performed subgroup analyses. As shown in Figure 5 A and B, a forest plot was also constructed to better demonstrate the decreasing trend of HRs in the high Cys‐C group among the subgroups. Elevated Cys‐C level was found to be significantly associated with poor OS in almost all subgroups.

## 4 DISCUSSION

The primary aim of this study was to analyze the association between preoperative serum Cys‐C levels and the oncological prognosis in patients with PRCC. Our findings demonstrated that patients whose serum Cys‐C levels were high had worse prognosis than those with low Cys‐C levels. After adjusting for other prognostic variables, preoperative Cys‐C was defined as an independent risk factor of OS in this particular subgroup. Therefore, we recommend preoperative Cys‐C levels to be a prospective predictor of patient survival, for clinicians.

Pretreatment Cys‐C levels have been shown to be an independent prognostic biomarker in various malignancies, such as nasopharyngeal, breast, and uroepithelial cancers.^14^, ^16^, ^21^, ^22^, ^23^ Kos et al. studied the prognostic value of serum Cys‐C levels among 345 patients with colorectal cancer and 125 healthy controls in a single center. They found that high Cys‐C levels were correlated with short survival times.^16^ Regarding RCC, Guo et al. initially evaluated preoperative Cys‐C levels of patients with RCC and found that elevated preoperative Cys‐C levels were shown to be associated with poor survival. They concluded that the testing of preoperative serum Cys‐C levels may be a convenient way to identify the poor prognosis in patients with RCC. In another study, Bodnar et al. proved the predictive significance of elevated serum Cys‐C levels in patients with metastatic RCC.^24^ However, these studies did not specifically analyze the effect of Cys‐C in patients with PRCC. The present study demonstrated that preoperative Cys‐C levels were significantly found to be corrected with age, comorbid symptoms, pathological grade, distant metastases, and histological subtype. In addition, multivariate analysis proved Cys‐C levels to be an independent risk factor for poor oncologic prognosis.

Cys‐C is widespread in the cells and tissues of various human tumors, although its expression is not consistent.^21^, ^25^, ^26^ It is also an inhibitor of cysteine proteases, such as histones (B, D, H, L, S), which play a regulatory role in cell proliferation, differentiation, and migration.^27^, ^28^, ^29^Previous studies have shown that Cys‐C inhibits the activity of histone proteases, a cysteine proteinase family, which facilitate the invasion and metastasis of cancer cells and is involved in other activities associated with tumor regression.^30^ Cys‐C plays a role in the immune response to damage due to tumors and is released in the serum by immune cells. In addition, the cystatin family induces nitric oxide, which is released from macrophages, and regulates cytosolic interleukins and cytokines in fibroblasts and T cells, which in turn regulate cell differentiation, proliferation, and bioactivity.^31^, ^32^ Therefore, elevated Cys‐C levels might indicate the onset of inflammatory and immune responses in humans, indirectly reflecting the malignant and destructive ability of tumors.

Serum Cys‐C levels have been proved to be a valuable biomarker to indicate renal function, and estimate eGFR more sensitively than that by serum creatinine levels.^33^ Cys‐C levels are also associated with impaired renal function.^34^ Furthermore, multifactorial analysis demonstrated that one of the independent predictors of survival in patients with PRCC was Cys‐C levels. Evidence suggests that renal insufficiency is significantly associated with high mortality after cancer treatment.^35^ In our research, we evaluated Cys‐C as the novel risk factor for prognostic survival in patients with PRCC. We found that patients with elevated preoperative serum Cys‐C levels were more likely to have higher creatinine levels and decreased renal function. The univariate analysis results also proved that eGFR was one of the important prognostic factors in patients with PRCC, consistent with previous findings.^36^, ^37^ Thus, the impact of Cys‐C on the prognosis of patients with PRCC might be attributed to its relationship with eGFR and renal function.

The present study findings showed that, in patients with PRCC patients, higher pretreatment Cys‐C levels were correlated with poorer clinical outcomes. Survival curves revealed a statistically significant discrepancy in survival between the two groups (*p* < 0.001). Similarly, we observed that the risk of postoperative mortality and OS decreased as Cys‐C levels increased, in the adjusted model. In this study, the cutoff values were determined using the X‐tile program. Notably, the optimal cutoff value for Cys‐C to predict tumor prognosis may vary with the tumor type, which might be related to tumor specificity, as well as to different sample sizes, survival endpoints, and follow‐up time. Conspicuously, our study incorporated time‐dependent ROC curves to estimate the predictive power of the factors and can be used to group continuous variables. In survival analysis, both, the factor values and the disease status change over time, whereas traditional ROC curve analysis treats individual factor values and disease status as smooth values, and time was not considered in the analysis. In this case, using the time‐dependent ROC is certainly a better alternative. Our findings are of interest from a clinical standpoint. Serum Cys‐C levels are one of the most common markers of renal function, routinely tested on hospital admission and area low‐cost and accessible preoperative predictor. Therefore, we recommend that preoperative Cys‐C levels should be considered by surgeons during preoperative evaluation or consultation when predicting patient survival post‐surgery. The opportunity provided by preoperative Cys‐C for earlier and more accurate prediction of tumor prognosis. In addition, preoperative Cys‐C levels may provide valuable suggestions for clinical anticancer treatment, or even the development of different treatment regimens, and monitoring of treatment effects. PRCC patients with high preoperative Cys‐C values should be followed up more aggressively and rigorously postoperatively.

Several limitations of this study warrant discussion. First, it is restricted by its retrospective character and confined to a single center, which may lead to a selection bias. Second, only patients with PRCC who received surgery were included in this study, which cannot represent other patients with PRCC who do not receive surgical treatment. Third, the primary endpoint of this study is OS, and further studies are needed to investigate the association of Cys‐C levels with tumor recurrence and tumor‐specific survival. Fourth, the cutoff value of Cys‐C was not based on the literature or previous studies, which might have an impact on the results. Fifth, since both eGFR and Cys‐C are factors in renal function, there is homology between Cys‐C and eGFR, which means that the effect of Cys‐C on the prognosis of PRCC may be influenced by eGFR. Finally, the sample size of this research was relatively small. Large prospective randomized trials are needed to validate the study findings.

## 5 CONCLUSION

This study explored the association between preoperative serum Cys‐C levels and prognostic OS in patients with PRCC. Serum Cys‐C levels can be considered a useful biomarker for preoperative screening of high‐risk patients, requiring adjuvant therapy. Patients with PRCC having high serum Cys‐C levels should be monitored more frequently postoperatively. Multicenter prospective cohort studies to further confirm the importance of Cys‐C in PRCC are warranted.

## CONFLICT OF INTEREST

The authors declare no conflict of interest.

## ETHICAL APPROVAL STATEMENT

This study was approved by the Ethics Committee of Shengjing Hospital of China Medical University. The clinical research registry number is 2021PS692K.

## AUTHOR CONTRIBUTIONS

SjL and SyL conceived and designed this research. SyL and SjL made contributions to the data gathering and analysis of this data. The original manuscript was drafted and revised by SyL. SjL prepared the figures and/or tables. The manuscript was edited by SjL and SyL. All authors have made contributions to this essay and endorsed the submitted version.

## Data Availability

The authors of this paper have provided the original data to support the conclusions of this paper without unnecessary reservations
